# Assessment of Artificial and Natural Transport Mechanisms of Ice Nucleating Particles in an Alpine Ski Resort in Obergurgl, Austria

**DOI:** 10.3389/fmicb.2019.02278

**Published:** 2019-10-04

**Authors:** Philipp Baloh, Nora Els, Robert O. David, Catherine Larose, Karin Whitmore, Birgit Sattler, Hinrich Grothe

**Affiliations:** ^1^Institute for Materials Chemistry, TU Wien, Vienna, Austria; ^2^Lake and Glacier Research Group, Institute of Ecology, University of Innsbruck, Innsbruck, Austria; ^3^Institute for Atmospheric and Climate Science, ETH Zürich, Zurich, Switzerland; ^4^Laboratoire Ampère, Environmental Microbial Genomics, École Centrale de Lyon, Université de Lyon, Écully, France; ^5^University Service Center for Transmission Electron Microscopy, TU Wien, Vienna, Austria

**Keywords:** ice nucleating particles, artificial snow, biological ice nucleation, alpine, particle transport

## Abstract

Artificial snow production is a crucial part of modern skiing resorts in Austria and globally, and will develop even more so with changing precipitation patterns and a warming climate trend. Producing artificial snow requires major investments in energy, water, infrastructure and manpower for skiing resorts. In addition to appropriate meteorological conditions, the efficiency of artificial snow production depends on heterogeneous ice-nucleation, which can occur at temperatures as high as −2°C when induced by specific bacterial ice nucleating particles (INPs). We aimed to investigate the presence, source and ice nucleating properties of these particles in the water cycle of an alpine ski resort in Obergurgl, Tyrol, Austria. We sampled artificial snow, river water, water pumped from a storage pond and compared it to samples collected from fresh natural snow and aged piste snow from the area. Particles from each sampled system were characterized in order to determine their transport mechanisms at a ski resort. We applied a physical droplet freezing assay [DRoplet Ice Nuclei Counter Zurich (DRINCZ)] to heated and unheated samples to characterize the biological and non-biological component of IN-activity. Bacterial abundance and community structure of the samples was obtained using quantitative PCR and Illumina Mi-Seq Amplicon Sequencing, and their chemical properties were determined by liquid ion-chromatography, energy dispersive X-ray spectroscopy (EDX) and scanning electron microscopy (SEM). The results show the flow of biological and inorganic material from the river to the slopes, an uptake of new microorganisms through the air and the piping, and possible proliferation or introduction of ice nucleation active biological particles in aged piste snow. Natural snow, as the first stage in this system, had the lowest amount of ice nucleation active particles and the least amount of biological and mineral particles in general, yet shares some microbial characteristics with fresh artificial snow.

## Introduction

A wide range of scientific research indicates a warming trend of the global climate, with the Alps especially prone (IPCC, 2014). Temperature rise in the 20th century is estimated to account for 1–2°C warming in alpine areas, in contrast to the 0.7°C predicted for the global mean, and this trend is accelerating (Auer et al., 2007). Austria, in particular, is projected to experience warming of more than 1.5°C by 2050 (Köppl et al., 2014). For the Alps, climate change is predicted to alter precipitation patterns and snow reliability (Uhlmann et al., 2009; Gobiet et al., 2014), which is expected to impact winter tourism (Elsasser and Bürki, 2002; Abegg et al., 2007). Climate change has reduced the spatial and temporal coverage of snow in skiing resorts, globally. In order to offset these reductions, ski resorts have turned to artificial snow production (Scott et al., 2003, 2006; Hennessy et al., 2008; Wolfsegger et al., 2008). In Austria, more than 67% of the skiing piste areas are equipped with artificial snow infrastructure (Köppl et al., 2014). Artificial snow production depends heavily on the weather conditions and also, to an extent, on the efficiency of the ice nucleation characteristics of the input substrate. In addition to the physical factors (temperature, humidity, droplet size) that control ice formation, the presence of ice nucleating particles (INPs) are required for ice formation to occur at temperatures above −35°C (Vali, 1971; Vali et al., 2015). INPs play an important role in the formation of clouds (Cziczo et al., 2013), where natural snow crystals originate. They therefore also impact cloud radiative properties, cloud lifetime and ultimately climate (Matus and L’Ecuyer, 2017). These particles can be of inorganic, carbonaceous or biological origin and have varying ice forming abilities (Murray et al., 2012; Kanji et al., 2017). The most efficient INPs are of biological origin, such as the bacterium *Pseudomonas syringae*, a well-studied plant pathogen found in a variety of ecosystems (Morris et al., 2013, 2007), and have been shown to form ice at temperatures as high as −2°C (Schnell and Vali, 1972; Maki et al., 1974; Vali et al., 1976; Möhler et al., 2007). *P. syringae* is also able to become airborne through aerosolization mechanisms (Pietsch et al., 2015) and showed a feedback mechanism with precipitation (Morris et al., 2017). The ice nucleating properties of *P. syringae* are linked to the gene INaZ with similar active genes also found in other bacteria that are not as well studied. The active compounds are located in the membrane (Green and Warren, 1985) and persist even when the organism is inactivated. Various existing technologies make use of this capacity, but their application in the environment is controversial (Ward and DeMott, 1989; Cochet and Widehem, 2000; Lagriffoul et al., 2010). For example, biological additives like Snomax^®^, originating from inactivated ice nucleating *P. syringae*, are prohibited for snow production in many countries, including Austria. Even when no additives are used for snow production, there are always INPs present in the water used and in the general environment. Their concentration has also been reported to be enhanced in precipitation and freshly fallen snow, indicating that certain active nucleators might trigger precipitation, and thus accumulate there (Christner et al., 2008a, b; Conen et al., 2017). Understanding the composition, efficiency and quantity of these INPs and their transport mechanisms is of interest for fundamental research in the field of geoscience and can be of interest for artificial snow production. Most research so far has focused on natural transport processes and detection of INPs therein. In a skiing resort, there are forced transport mechanisms where water is filtered, stagnant, transported through pipes and aerosolized. In this study we investigate for the first time how microbial as well as non-microbial INPs are introduced, distributed and persist in such a system and assess their impact on the overall ice nucleation ability of the water.

The microbial diversity in alpine soils strongly depends on the spatial and temporal distribution of snow cover (Zinger et al., 2009), indicating that snow microbes represent an important inoculum for underlying soil (Lazzaro et al., 2015a, b) and consequently other adjacent habitats. Microbes are of special interest since they can proliferate under suitable conditions in contrast to non-microbial particles, thus multiplying their impact on the system. Artificial snow alters the microbial composition of underlying soils due to its higher density and late melting, implying shorter vegetation periods (Newesely and Cernusca, 1999; Rixen et al., 2004, 2008). The chemical composition of the water used for artificial snow production can lead to an altered input of chemical components into the ecosystem relative to natural snow (Jacobi et al., 2006; Rixen et al., 2008), and ultimately become a major nutrient input source in high alpine elevations (Johannessen and Henriksen, 1978; Jones, 1999; Jones and Harrison, 2004). It is currently unknown how the microbial diversity and abundance of artificial snow compares to the source water of the artificial snow, natural snow and piste snow. To investigate the properties of artificial snow compared to its source material and identify potential natural sources of INPs, but also to assess the change of microbial composition in an artificial snow production cycle, we conducted a microbiological, chemical and ice nucleation activity characterization of fresh snow, piste snow, artificial snow and its water sources.

## Materials and Methods

### Study Site and Sample Collection

**FIGURE 1:**
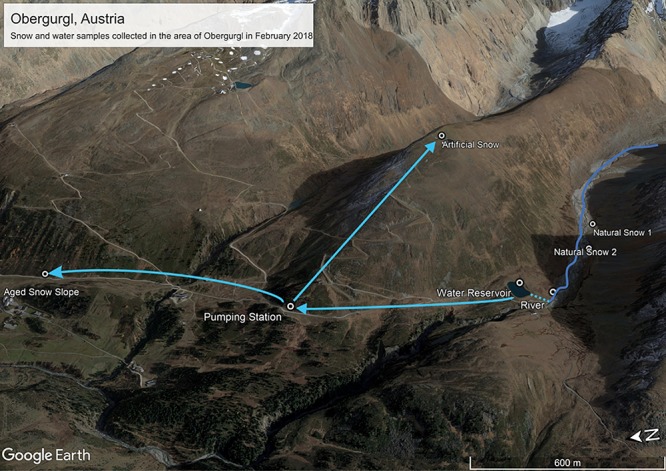
Sampling sites and connection of the water system in Obergurgl, Tyrol, Austria. Adapted from image source: “Obergurgl” 46°51′4.51″N and 11° 1′22.75″E. Google Earth pro. October 19, 2017. Accessed January 27, 2019.

Samples were collected on the 22.02.2018 (all samples apart from aged piste snow) and the 23.02.2018 (only aged piste snow) in Obergurgl, Austria (see Figure 1 and Table 1). Obergurgl is a large skiing area in the inner Ötz Valley, south-west Tyrol (1.800–3.080 m a.s.l.). Fresh precipitation fell on the 22.02.2018, with air masses transiting from northern Africa, the Mediterranean and south-eastern Europe in the 120 h before arriving in Obergurgl (see Supplementary Figure S2 for *Hysplit* calculation). The fresh snow samples were collected in Rotmoostal (∼2275 m a.s.l.), a valley that connects to the Rotmoosferner, a glacier mass southeast of Obergurgl. The Rotmoosache river flowing through this valley is fed by glacial melt and springs with a strong seasonal change in water masses and composition, as characteristic for high alpine glacial fed rivers. An artificial pond (Schönwies water reservoir, ∼2270 m a.s.l.) fed by the Rotmoosache river is located at the end of the valley and is operated by the Obergurgl lift company. Water is pumped from this pond to a pumping station (Steinmann pumping station, ∼2250 m a.s.l.) north of the reservoir that supplies several locations for artificial snow production. One of these locations is the Hohe Muth (∼2640 m a.s.l), a mountain top from which the artificial snow samples were collected. Snow samples (approx. 1 L meltwater each) were taken with a 10 times precontaminated stainless steel shovel (ROTH) into sterile Nasco Whirl-Paks (ROTH). One river sample and triplicates of water from the collection pond were collected in 1 L polypropylene bottles (Nalgene) that were pre-rinsed with 15% HCl, three times milli-Q water and three times pre-contaminated with on-site sample water. The snow samples were collected as triplicates in a triangle with approximately 2 m distance between the spots. For the fresh snow samples, only the first 100 mm of the snow layer were collected. The collected artificial snow was produced just for this study. A conventional snow cannon (Demac Lenko, Titan 2.0) was used to produce snow for 30 min that was subsequently collected. Lifts were already closed at this time and the sampling area was undisturbed. For the artificial snow samples, only the uppermost 20 mm were collected. The water pipes for the artificial snow production were empty beforehand (to prevent freezing damage) and filled with water prior to the operation. Figure 1 depicts the connection of the water system. The pumping station pumps the water from the reservoir into the water pipes on the mountain used for snow production. Under standard operating procedures in the federal state of Tyrol, the water has to pass an UV-disinfectant unit in the pumping station, but the unit was deactivated for this study. UV-purification of water for artificial snow production is only mandatory in the federal state of Tyrol, but not in other parts of Austria nor globally. Therefore, in order to render this study relevant for artificial snow production at a global scale, we chose to use untreated water.

**TABLE 1:** Sampling table.

**Sample**	**Description**	**Sampling date/time**	**Means of collection**	**GPS**
Steinmann 1	Pumping station pond	22.02.2018, 09:30	Bottle	46.856238,11.019306
Steinmann 2	Pumping station pond	22.02.2018, 09:32	Bottle	46.856238,11.019306
Steinmann 3	Pumping station pond	22.02.2018, 09:34	Bottle	46.856238,11.019306
OBA 61	River	22.02.2018, 14:30	Bottle	46.8462314,11.0180409
S_1_1	Fresh snow 1	22.02.2018, 12:00	Whirl-Pak	46.8418793,11.0252594
S_1_2	Fresh snow 1	22.02.2018, 12:20	Whirl-Pak	46.8418793,11.0252594
S_1_3	Fresh snow 1	22.02.2018, 12:40	Whirl-Pak	46.8418793,11.0252594
S_2_1	Fresh snow 2	22.02.2018, 13:20	Whirl-Pak	46.8431900,11.0222406
S_2_2	Fresh snow 2	22.02.2018, 13:30	Whirl-Pak	46.8431900,11.0222406
S_2_3	Fresh snow 2	22.02.2018, 13:50	Whirl-Pak	46.8431900,11.0222406
S_3_1	Artificial snow	22.02.2018, 17:30	Whirl-Pak	46.8490396,11.0284536
S_3_2	Artificial snow	22.02.2018, 17:40	Whirl-Pak	46.8490396,11.0284536
S_3_3	Artificial snow	22.02.2018, 17:50	Whirl-Pak	46.8490396,11.0284536
S_4_1	Aged natural piste snow	23.02.2018; 8:00	Whirl-Pak	46.8658371,11.0262297
S_4_2	Aged natural piste snow	23.02.2018; 8:10	Whirl-Pak	46.8658371,11.0262297
S_4_3	Aged natural piste snow	23.02.2018; 8:20	Whirl-Pak	46.8658371,11.0262297

The aged piste snow samples were collected in the morning before the lifts opened after being exposed to 1 day on an operating ski piste (20 h after fresh snow samples were collected). For these samples, the uppermost 200 mm were collected to include aged and not only fresh snow. The piste was compacted by the grooming that takes place every evening and morning. In contrast to the other snow samples, the aged piste samples underwent an unknown number of freeze-thaw cycles. The location of aged piste samples was also in closer proximity to the village of Obergurgl and therefore, more exposed to anthropogenic pollution. The water samples at the pumping station were collected from a valve on the pipes that pump the water up to the snow cannons. Water from the river that was used as a source for the snow machines was collected close to an inlet that transports it into the water reservoir. The reservoir itself was not sampled due to safety reasons.

### Ice Nucleation Assays

The ice nucleation experiments were performed using the DRoplet Ice Nuclei Counter Zurich (DRINCZ) as described by David et al. (2019). Briefly, DRINCZ uses a temperature controlled ethanol bath (Lauda ProLine RP 845) to cool a partially submerged well plate (732–2386, VWR) that holds 96 50 μL aliquots of a sample, to temperatures between 0 and −25°C at a cooling rate of 1°C min^–1^. A camera (Microsoft Lifecam HD-3000) monitors the light transmission through the aliquots every 0.25°C. Upon freezing, the light intensity through the aliquot changes, allowing the camera to detect the freezing temperature of an aliquot within ±0.9°C (David et al., 2019). All of the presented freezing results are background corrected using the same technique described in David et al. (2019) as the sampling procedure was found to have no impact on the background freezing of the DRINCZ setup. The concentration of INPs in the melted snow or water samples as a function of temperature (*n*_*mw*_(*T*)) is calculated following Vali (1971) and Vali et al. (2015):

$$n_{\text{mw}}\,\left(T\right)=\frac{-\text{ln}\,\left(1-F\,F\,\left(T\right)\right)\cdot D\,F}{V_{a}}\times 1,000,000$$

where *FF*(*T*) is the frozen fraction of aliquots as a function of temperature (°C), *V*_*a*_ is the volume of an aliquot (50 μL) and *DF* is the dilution factor of the sample. The multiplication by 1,000,000 converts the results to L^–1^ of snow melt or water.

All samples were stored frozen at −20°C until and after measurement with DRINCZ in order to avoid potential changes in the INP activity (Stopelli et al., 2014). In order to determine the contribution of biological INPs to the observed freezing ability of a sample, one of the previously measured triplicates from each sample type was placed frozen into an oven (Binder, APT. line FD115) at 95°C for 20 min and then directly transferred and measured in DRINCZ. It is important to note that the aged piste snow sample was only heated for 15 min and the impacts of this are discussed further in section “Biology Drives Highly Active Ice Nucleation in Snow and Water Samples” and Supplementary Figure S9. Heating the samples distinguishes between microbial and non-microbial ice nucleation (Christner et al., 2008a), since both inorganic INPs and ice nucleating macromolecules such as those from birch remain unaltered at the temperatures used (Sullivan et al., 2010; Pummer et al., 2012; Zolles et al., 2015; Felgitsch et al., 2018).

### Chemical Information and Imaging

#### Aquatic Chemistry

A portion of each sample for chemical analysis was filtered through a precombusted glass fiber filter (MN GF-5, Marcherey–Nagel, 25 mm). Anions and cations were measured by ion-chromatography (ICS-1100 resp. ICS-1000 Dionex). For determination of dissolved organic carbon (DOC) (Shimadzu TOC-CPH) and dissolved nitrogen (DN) (Shimadzu TNM-1) samples were acidulated with 200 μL 2N HCl to reach a pH of 1.5–2.

#### Scanning Electron Microscopy

For scanning electron microscopy (SEM) imaging, 3 mL of the samples were freeze dried (Christ Alpha 1–4 LDplus; 24 h at 1 mbar and −55°C and 4 h at −55°C and 10^–2^ mbar) and the residues were dispersed in 200 μL of ultrapure water (produced with Millipore^®^ SAS SIMSV0001). From this concentrated solution, droplets were deposited with a micropipette on a clean aluminum sample holder and dried under a constant nitrogen gas flow until the next droplet was deposited. Ten droplets were accumulated per sample to produce a layer of observable residues. With this method, the residues show the solid particles present in the water samples and also solidified products of the solved substances from the water. Once completely dry, the samples were sputtered with a 4–6 nm Au/Pd 60:40 layer and transferred to the SEM (FEI Quanta 250 FEG) which operates at a vacuum of 10^–6^ mbar. Images were taken with an accelerating voltage of 5 kV.

#### Energy Dispersive X-Ray Spectroscopy

The SEM is equipped with and energy dispersive X-Ray spectroscopy (EDX) unit (EDAX SDD Octane Elite 55) to allow for elemental analysis of the samples. Measurement EDX spectra of agglomerations and particles spotted on the SEM images were recorded. Spectra and images for EDX were recorded with a voltage of 20 kV.

### Microbial Analysis

#### DNA Extraction and Sequencing

The molten snow and water samples (1 L each) were filtered through a 0.2 μm polycarbonate filter (47 mm, Isopore). DNA was extracted from the filters using DNeasy Power Water extraction kit (Qiagen) following the provided protocol. Amplification, library preparation and sequencing was done at the Environmental Microbial Genomics group at the Laboratoire Ampère (ECL Lyon, University of Lyon, France). Community diversity was targeted: the V3–V4 region of the bacterial 16S rRNA SSU gene was amplified using 338F/518R primers and the fungal internal transcribed spacer (ITS) regions were amplified with primer pair ITS2-ITS4. Raw sequences were stored at NCBI BioProject database under Project ID PRJNA534428.

#### Bioinformatics

Forward and reverse reads were merged using *vsearch* (Rognes et al., 2016). Sequences were quality filtered and assembled in *QIIME pipeline* (Caporaso et al., 2010). Chimeras were removed using *UCHIME* (Edgar et al., 2011) with closed reference and *de novo* approach. Operational taxonomic units (OTUs) were assembled at 97% similarity using *vsearch* classifier at default settings (Rognes et al., 2016) and blasted against SILVA 132 database (Yilmaz et al., 2014; Glöckner et al., 2017) for bacteria, and UNITE 7.2 database (Kõljalg et al., 2013) for fungi. Singletons were removed. Negative control OTUs from the kit blank were subtracted for all samples (see Supplementary Table S5 and Supplementary Figure S3 for sequence statistics, removed blank genera and rarefaction curves). Statistical analyses were done in *R* (R Core Team, 2015) using the *phyloseq* (McMurdie and Holmes, 2013), *vegan* (Oksanen et al., 2018), and *ggplot* (Wickham, 2009) packages. OTUs were merged into genus level for all analyses apart from alpha diversity to account for species length polymorphisms in fungal ITS regions below genus level (Gomes et al., 2002) and uncertainties in bacterial taxonomy below genus level.

Bray–Curtis distance was calculated on third-root transformed datasets of 97% most common OTUs on genus level and ordinated with principal coordinate analysis (PCoA). Datasets were not rarefied, according to Weiss et al. (2017).

Obtained samples were blasted (blast version 2.8.2) against a nucleotide database of organisms containing the ice nucleation protein (search for “ice nucleation protein”), created from NCBI^1^, with a threshold of 97% similarity. The obtained reads were filtered for matches of 400 basepairs and longer. Ice-nucleation activity was not verified in laboratory studies for all the bacteria in the blast database that was generated. It is possible that the gene is inactive in some organisms (Miller et al., 2016) and not all cells containing an active ice-nucleation protein exhibit ice-nucleation activity at all times or temperatures (Lindow et al., 1982, 1989).

#### Quantitative PCR

To estimate the abundance of bacteria and fungi, both 16S rRNA genes [primer 338F/518R (Øvreås and Torsvik, 1998)] and 18S rRNA genes [primer set FR1/FF390 (Chemidlin Prévost-Bouré et al., 2011)] were quantified by qPCR using Quantifast 2X SYBRGreen dye (Qiagen). Non-template controls were subtracted.

## Results

### Ice Nucleation Activity

**FIGURE 2:**
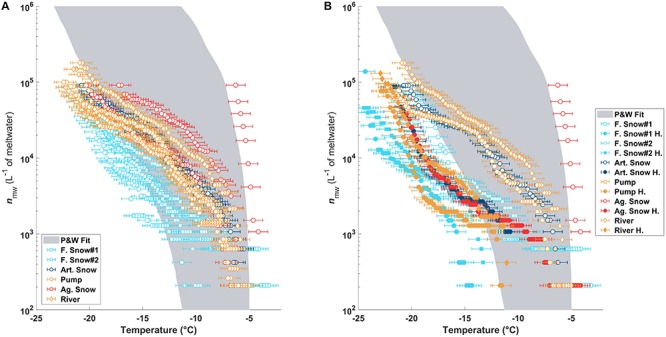
**(A)** The *n_mw_*(*T*) as measured in DRINCZ for all triplicate of each sample type. The gray shaded region is the fit for previously published *n_mw_*(*T*) from precipitation samples (Petters and Wright, 2015). Panel **(B)** is the same as **(A)** but compares one of the triplicates before (open circles) and after heating (filled circles).

The *n*_mw_(*T*) of the sample types and their triplicates are shown in Figure 2A and are consistent with previously observed values in precipitation samples as summarized in Petters and Wright (2015) (shaded gray region). The river, pond/pump, and artificial snow samples grouped closely. The highest concentration of INPs at high temperatures and the largest heterogeneities were found in the aged slope snow. In contrast, the natural snow samples showed the lowest activities of all samples and extended below the previously reported INP concentrations from precipitation (Figure 2A; Petters and Wright, 2015).

After heating, the most efficient INPs in all of the samples were deactivated (Figure 2B), shifting the curves to lower temperatures outside of the Petters and Wright fit area. Furthermore, when comparing the temperature required for 50% of the aliquots to freeze (T50), the temperatures became very similar to those before heating (see Table 2).

**TABLE 2:** T50 of native and heated samples and their difference.

**Sample**	**T50 (°C) native**	**T50 (°C) heated**	**ΔT50**
Fresh snow 1	−17.27	−21.70	−4.43
Fresh snow 2	−18.87	−21.16	−2.29
River	13.75	−20.31	−6.20
Pumping station	−12.91	−20.50	−13.69
Artificial snow	−13.12	−19.33	−6.55
Aged piste snow	−5.32	−19.02	−7.58

**TABLE 3:** Main elements contributing to composition of samples assessed by EDX.

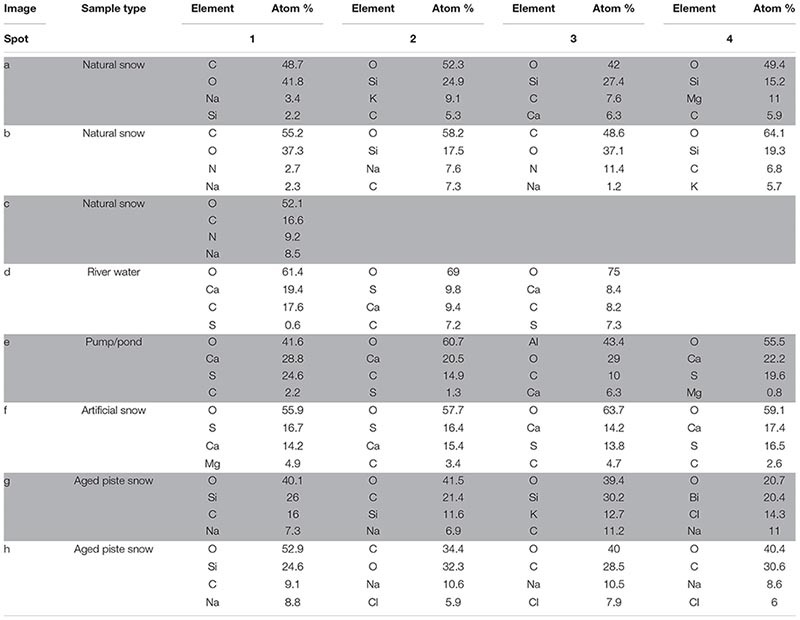

The highest change in T50 (ΔT50) occurred in the aged piste snow (ΔT50: −13.7°C) as shown in Table 2. Pond, river and artificial snow showed very similar T50 and ΔT50 values, consistent with *n*_mw_(*T*), while fresh snow samples showed the least sensitivity to heating.

### Chemical Information and Imaging

#### Aquatic Chemistry

**FIGURE 3:**
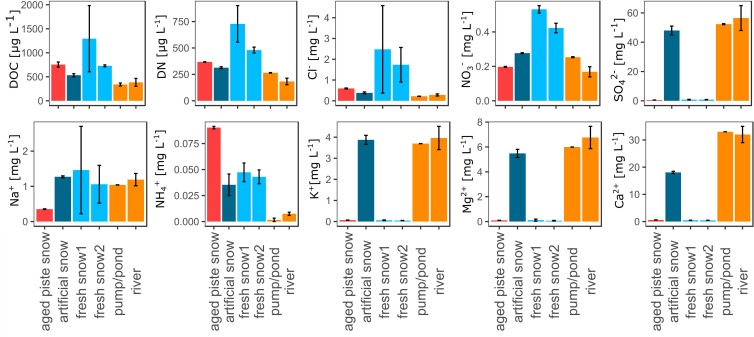
Dissolved organic carbon (DOC), dissolved nitrogen (DN), chloride (Cl**^–^**), nitrate (NO_3_**^–^**), sulfate (SO${}_{4}^{2-}$), sodium (Na^+^), ammonium (NH_4_^+^), potassium (K^+^), magnesium (Mg^2+^), and calcium (Ca^2+^), in μg resp. mg per L water or molten snow.

The aquatic chemistry among the samples was significantly different for most parameters as seen in Figure 3 and Supplementary Tables S3, S4. The fresh snow samples and the aged snow showed the highest values for DOC, DN, and Cl^–^, whereas the samples from the river water cycle (river, pumping station, artificial snow) showed lower values. NO_3_^–^ was also highest in the fresh snow samples, but not in the aged piste snow. SO_4_^2–^, K^+^, Mg^2+^, and Ca^2+^ were all low in the fresh and aged snow samples. Na^+^ values were significantly lower for the aged piste snow samples in comparison to all other samples. NH_4_^+^ was significantly higher in the aged snow samples, in medium range for artificial and fresh snow and lower for the river and pumping station samples. The mean error revealed that the fresh snow samples (especially fresh snow2) were the most heterogeneous in the overall analysis. See Supplementary Table S6 for aquatic chemistry values.

#### SEM and EDX

**FIGURE 4:**
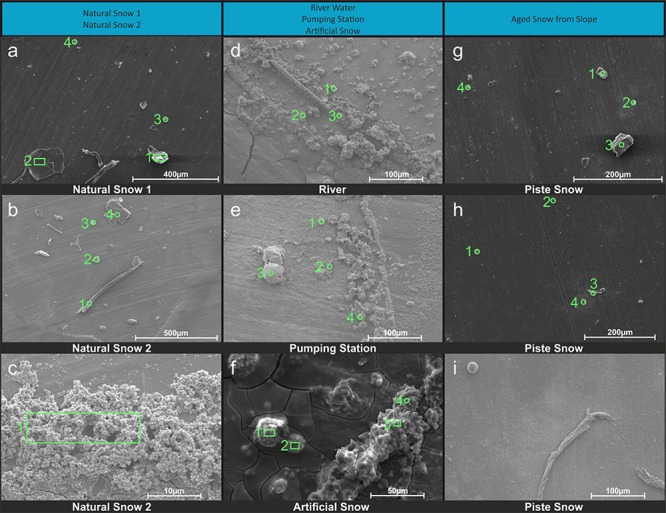
SEM images of snow and water sample residues. Green circles indicate where EDX single spot spectra were taken (1 mm diameter) and green rectangles indicate where larger areas were scanned for a spectrum. A column represents a group of related samples with the black bar under each image indicating which specific sample is shown. Images **(a–c)** are natural snow samples, **(d–f)** are samples derived from the riverwater and **(g–i)** are different spots on the aged snow. The associated elemental information is summarized in Table 3.

The SEM images and its EDX spectra information were grouped into three main categories based on their morphological similarities represented by the columns in Figure 4. Due to the method used, former solved substances from the water samples are also visible the image, in addition to particles present at sampling. This has to be taken into consideration especially for samples from the river water, as they are much higher in dissolved ions known to form mineral precipitates as shown in section “Aquatic Chemistry.”

##### Natural snow samples

In natural snow (Figures 4a–c), various particle classes were present. Biological materials like fibers (b1) and presumably insect parts (a1), mineral particles of different morphology (a2, a3, a4, b2, b4) and agglomerations of small particles (c1) were identified. Biological particles were identified by their morphology and carbon content. Plant fibers, as observed in b1, were identified based on their distinctive morphology and fine structure, in combination with a high carbon content. Particle a1 was identified as either a heavily weathered insect part or a carbonate mineral. The mineral particles in these samples consisted mainly of quartz and its varieties, as shown by the high content of Si and O. On image c, an agglomeration of particles in the 1 μm size range was scanned. A thin ring of these agglomerations was observed on the borders of the sampling area. Elemental analysis showed high contents of O, C, and N. With the lack of crystalline features, the agglomerations could be made up of dried biological material such as bacteria. The general amount of material in the natural snow samples was lower compared to the samples from other sources.

##### Samples influenced by river water

The samples influenced by river water all bore similar features. The sampling area was full of residual material with distinctive crystalline features as well as a thin amorphous film that covered most of the sample. The film itself is clearest in Figures 4d,f, where cracks in it were present. Unfortunately, the film was too thin to gain useful information on it by EDX. All analyzed spots in river water (Figure 4d) showed mineral particles with variable Ca and S concentrations. Particles with a high Ca and low S content had a more columnar appearance (d1, d3), whereas particles with a higher S content exhibited a more globular appearance with needles as fine structures (d3). The pumping station sample (Figure 4e) contained more globular particles (e1, e4), high in O, S, and Ca, similar to those in Figure 4d. Small scale oval structures were found with a high Ca, C, and O content. The relatively smooth rounding and lack of crystalline features points to biological material. The particle at e3 showed very high contents of Al. Therefore, the Al was exceptionally included in the elemental analysis. The artificial snow sample (Figure 4f) was produced with water from the pumping station. The analyzed spots all show a very similar composition in EDX analysis with major parts of O, Ca, and S. The spots with higher amounts of S (f1, f2) reveal a more globular morphology. A visible film covers the sample, yet was too thin to obtain elemental information.

##### Aged snow from the skiing piste

The snow collected from the piste showed a lower coverage of residue in the sample area than the samples from the river water. The particles found in the piste samples, however, differ from the natural snow samples. Spot g1, g2, and g3 show particles with a high oxygen and silicon content, but also relatively high amounts of carbon. This points to organic compounds absorbed on quartz or other silicate mineral particles. On spot g4, a particle had an elemental composition of oxygen, bismuth, chlorine and sodium. In image h on spot h1 is a particle similar to those in image g with high amounts of O and Si in very accurate stoichiometry for SiO_2_, which points to the particle being composed of quartz and/or feldspar_._ The carbon signal is probably present due to absorbed organic material from different sources. The other spots in image h all show organic material with absorbed sodium chloride. In image i a natural fiber is shown and a particle that looks like an egg of an insect or aquatic organism. No EDX was carried out on these particles.

### Microbial Analysis

#### Microbial Abundances

**FIGURE 5:**
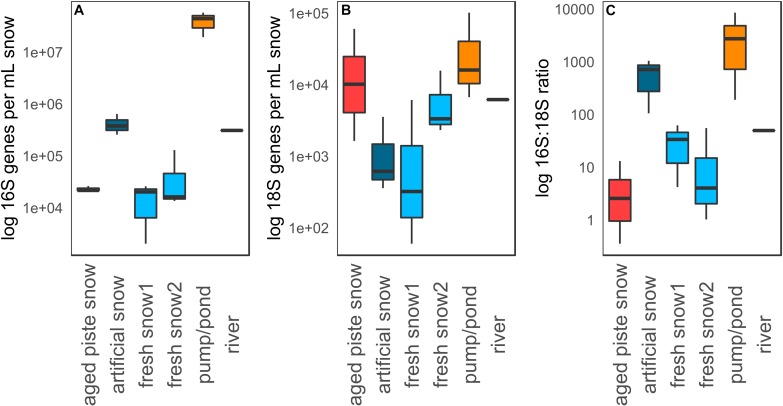
**(A)** qPCR data for 16S genes, **(B)** 18S genes, and **(C)** 16S to 18S ratio per mL water or molten snow.

Figure 5 shows qPCR data of 16S genes, 18S genes and 16S–18S ratio per mL meltwater in the different snow types that were sampled. The highest abundance in both 16S and 18S rRNA genes mL^–1^ was detected in the pond (16S: 5.78 × 10^7^ genes mL^–1^, 18S: 1.05 × 10^5^ genes mL^–1^), while the lowest was in fresh snow 1 (16S: 2.04 × 10^3^ genes mL^–1^, 18S: 6.02 × 10^1^ genes mL^–1^). Significant differences in 16S gene abundance (Kruskal–Wallis *p* = 0.03) were detected among sample types, with the pond abundance being significantly different from aged piste snow (Dunns *p* = 0.013), and fresh snow 1 and 2 (Dunns *p* = 0.002, *p* = 0.004) and artificial snow being significantly different from fresh snow 1 and 2 (Dunns *p* = 0.036, *p* = 0.024). There was no overall significant difference between sample types for 18S rRNA abundance (Kruskal–Wallis *p* = 0.13), but the pond water was significantly different from artificial snow (Dunns *p* = 0.013) and fresh snow1 (Dunns *p* = 0.006). Fresh snow1 also differed significantly from aged piste snow (Dunns *p* = 0.043).

The highest 16S:18S rRNA gene ratio was detected in the pond water (8.59 × 10^3^:1), while the lowest was detected in aged piste snow (2.58 × 10^0^:1). Ratios of sample types were significantly different from each other (Kruskal–Wallis *p* = 0.03). Aged piste snow was significantly different to artificial snow (Dunns *p* = 0.008) and the pond (Dunns *p* = 0.002), artificial snow was significantly different to fresh snow 2 (Dunns *p* = 0.024), and the 16S:18S ratio of the pond water was significantly different to fresh snow 1 and 2 (Dunns *p* = 0.036, *p* = 0.008), see Supplementary Tables S3, S4.

#### Microbial Diversity

##### Alpha diversity

There were 5874 bacterial and 992 fungal OTUs in the dataset after blank removal. Bacterial richness (Chao1) and evenness (Shannon) was significantly different between groups (Kruskal–Wallis *p* = 0.03 resp. *p* = 0.02), for fungi only the richness (Chao1) differed significantly (Kruskal–Wallis *p* = 0.03). The highest bacterial Chao1 value was found in river water and artificial snow, the lowest in fresh snow1. Aged piste snow was different from artificial snow (Dunns *p* = 0.003), fresh snow2 (Dunns *p* = 0.029) and river (Dunns *p* = 0.01). Artificial snow was significantly different from fresh snow1 (Dunns *p* = 0.024) and the pond/pump (Dunns *p* = 0.016), and for river and fresh snow1 (Dunns *p* = 0.039) and pond/pump (Dunns *p* = 0.030). Bacterial Shannon index was highest for the river and lowest for the pond/pump. Highest fungal Chao1 and Shannon indices were found in aged piste snow and the lowest in pond/pump water. Pond/pump fungal Chao1 and Shannon were significantly different from piste snow (Dunns *p* = 0.001) and fresh snow1 and 2 (Dunns *p* = 0.029, 0.019), artificial snow and piste snow also differed (Dunns *p* = 0.012) (see Supplementary Tables S3, S4 and Supplementary Figure S6).

##### Beta diversity

**FIGURE 6:**
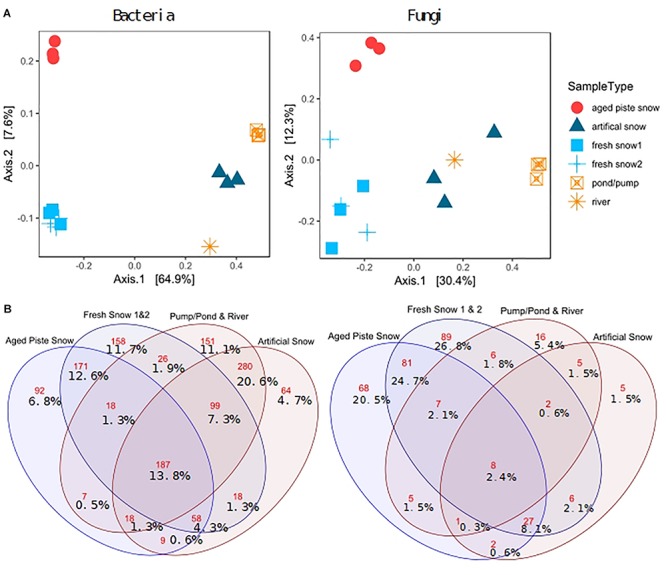
**(A)** PCoA on Bray–Curtis distance of third-root transformed 97% most common genera explained variability in brackets, **(B)** Venn diagram of shared and unique genera (red numbers: absolute genera, %values: % genera of total genera).

Figure 6A shows a PCoA on genus level for bacteria and for fungi. This method of multidimensional scaling finds the main axes to explain dissimilarities in a multivariate dataset using eigenvectors. The first two axes explain 72.5% of bacterial and 42.7% of fungal dissimilarity.

The sample types were significantly different in diversity for bacteria (ANOSIM: *R* = 0.962, *p* = 0.001, ADONIS: *p* = 0.001, *R*^2^ = 0.868) and for fungi (ANOSIM: *R* = 0.7498, *p* = 0.001, ADONIS: *p* = 0.001, *R*^2^ = 0.574). The PCoA revealed a clear separation between samples of natural snow formation and of pond sources, for both bacteria and fungi. For fungi, the artificial snow and river samples clustered closer to the natural samples than for the bacteria (see Supplementary Table S1 for statistics, Supplementary Figure S1 for abundant phyla and classes and Supplementary Figure S6 for clustering).

Pairwise PERMANOVA comparison revealed significant differences in bacterial composition of fresh and piste snow (PERMANOVA *p* adj. = 0.05), fresh snow and pond water (PERMANOVA *p* adj. = 0.3) and fresh vs. artificial snow (PERMANOVA *p* adj. = 0.05), as well as piste snow vs. pond water (PERMANOVA *p* non-adj. = 0.34). For fungi, pairwise comparison revealed significant differences in fresh snow and pond water (PERMANOVA *p* adj. = 0.024) and fresh and artificial snow (PERMANOVA *p* adj. = 0.05). Unadjusted *p*-values were also significant for piste vs. fresh snow (PERMANOVA *p* = 0.013) and piste snow vs. pond water (PERMANOVA *p* = 0.031).

Artificial snow contained 4.7% unique bacterial genera. Natural snow sources (fresh snow and piste snow) contained 72% of all unique fungal genera, with only 2.4% shared by all sample types. In contrast, 13.8% of bacterial genera were present in all sample types and 31% of bacterial genera were unique to fresh and piste snow, while 36% was unique to artificial snow and pond/pump and river water. Artificial snow contained 8.1% (i.e., 27) of genera in common with fresh snow and piste snow, but only 1.5% with pond/pump and river water (see Supplementary Table S2 for the shares of most abundant bacterial phyla and fungal classes in the sample types).

#### Relative Abundance of Genera Known to Encode Ice Nucleation Proteins

**FIGURE 7:**
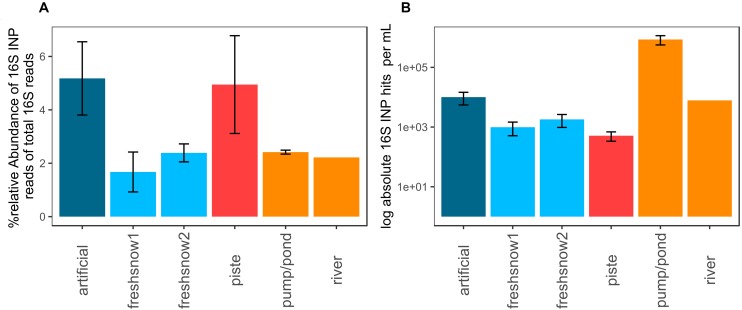
**(A)** % relative abundance of 16S rRNA gene reads with the closest hit to organisms containing an ice nucleation protein, **(B)** log absolute 16S potential ice nucleation-protein concentration per mL molten snow or water (mean by sample type, error bars: standard deviation of mean).

The artificial snow and piste snow contained the highest relative share (5.5%) of reads allocated to bacteria possessing an ice nucleation protein. Fresh snow, pond/pump and river snow contained lower amounts (1.6–2.2%, Figure 7A). Fungi had no hits.

When corrected by 16S rRNA gene abundance approximated using qPCR (Figure 7B), pond water reached the highest concentrations of potential biological ice-nucleation protein containing bacteria per mL (8.6 × 10^5^ genes mL^–1^), while artificial snow and river water had similar values (1 × 10^4^ genes mL^–1^, 7.8 × 10^3^ genes mL^–1^). Fresh snow 1 and 2 (1.8 × 10^3^ genes mL^–1^, 9.9 × 10^2^ genes mL^–1^) and aged piste had lower numbers, with piste snow displaying the lowest ice-nucleation protein bacteria abundance (5.1 × 10^2^ genes mL^–1^).

**FIGURE 8:**
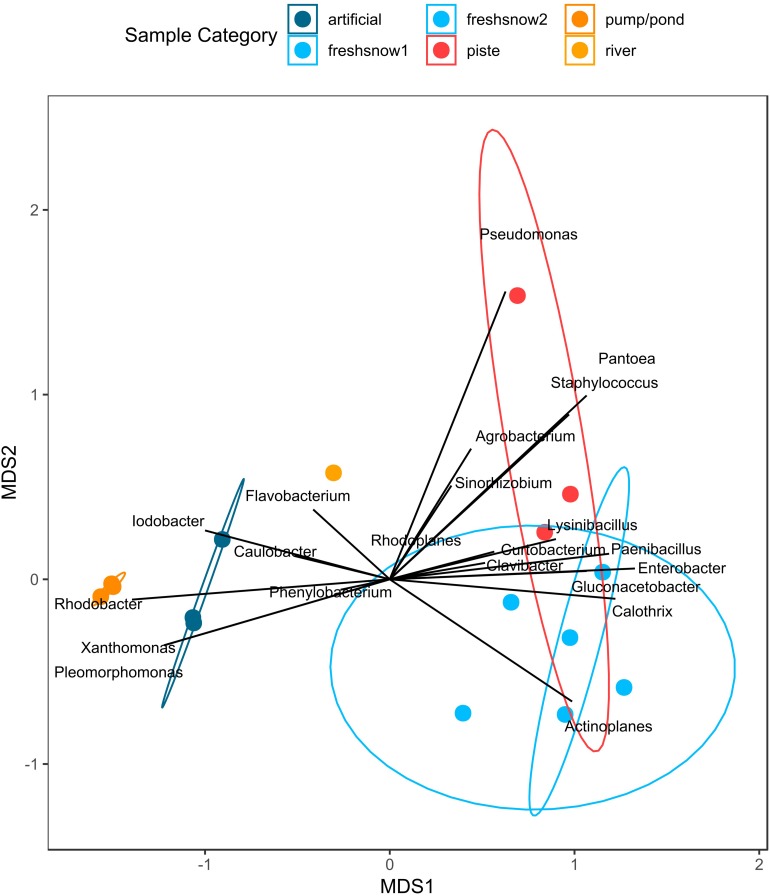
NMDS of relative abundance of bacteria containing an ice nucleation protein by sample type.

Figure 8 shows two clusters of bacterial genera along the MDS1 axis (explaining most of the dissimilarity), one being associated with presence in artificial snow and its sources (e.g., *Iodobacter*, *Xanthomonas*, *Rhodobacter*, *Pleomorphomonas*), the other genera cluster to the sample types of fresh snow and piste snow (e.g., *Actinoplanes*, *Paenibacillus*). Piste snow was distinguished on the MDS2 axis by *Pseudomonas*, *Pantoea*, and *Staphylococcus*. When ordinated on the highest resolution (i.e., strains) there is a difference in closest hits to ice nucleation protein carrying bacteria strains within the different sample types (see Supplementary Figure S8 for closest strain hits).

## Discussion

### Ice Nucleation Activity Explained by Chemistry of the Samples

The aquatic chemistry and elemental analysis from residues by EDX provided information on the chemical composition of substances in the collected samples. Since the range of ice nucleation altering materials spans many substances, from inorganic to organic and biological particles (Kanji et al., 2017), it is difficult to identify a single substance and its impact, especially in field samples where many of these substances occur, often intermixed. It is however possible to narrow down certain classes of INPs that can affect the ice nucleation ability of water samples. Mineral particles could be identified in all samples, which is to be expected as they make up the largest share of aerosol particles (Cziczo et al., 2004; Pratt et al., 2009) and are the dominant fraction in the alpine river water as well. The mineral particles brought into the system by snow precipitation likely vary from the particles in the river water, which represent the local geology and the dust particles in the area due to selection processes taking place when transported in the atmosphere (D’Almeida and Schütz, 1983; Murray et al., 2012; Knippertz and Stuut, 2014). In the fresh snow samples and the aged snow samples, particles rich in Si were dominant. This is an indicator that quartz or feldspar particles, important ice nuclei (Atkinson et al., 2013; Zolles et al., 2015), might be active in natural snow precipitates. However, the effect of this mineral aerosol on the ice nucleation activity of the river water and artificial snow production is likely negligible, since the overall number of particles transported by a glacial fed alpine river is magnitudes larger (Milner and Petts, 1994; Ward, 1994; Gurnell et al., 1996) than the particles brought into the system by precipitation (Boose et al., 2016). The mineral composites present in the river was tracked to the pumping station and to the artificial snow, as shown by the aquatic chemistry and SEM/EDX results and indirectly through the DRINCZ results. A clear grouping of these samples was observed with all methods. The mineral particles native to the river water were therefore identified as the main source of inorganic INPs in the artificial snow production cycle. EDX shows O, Ca, S and C as main components indicating sulfates, carbonates, and oxides. The globular structures with needles that are high in O, S, and Ca content seen in the SEM images are an indicator for dehydration and hydration processes of gypsum (Azimi and Papangelakis, 2011) and have also been shown to have an effect on ice nucleation (Grawe et al., 2018). The other columnar structures with strong O, Ca, C signals were likely made of calcium carbonates, which are among the lesser active INPs (Atkinson et al., 2013). No strong silicate signals were found in the residues from the river water by EDX, however, the absence of silicates in the river water samples was unlikely due to the geology in the area (Tropper and Recheis, 2003), and silicon rich particles might be covered by calcium carbonates and sulfates during the freeze drying process. The mineral particles found in the aged snow were more similar to those found in the fresh snow samples. This would suggest that the aged snow that was sampled is natural, rather than artificial. Artificial snow is predominately used at the start of the season (November/December) in Obergurgl and at the time of sampling, most of the piste is composed of natural snow. The particles in the aged snow may be exposed to stronger weathering by chemical compounds through environmental factors and anthropogenic pollution, which can affect the ice nucleation ability (Reitz et al., 2011; Zolles et al., 2015). This is especially relevant since the piste undergoes a number of freezing and thawing cycles which promotes diffusion and the snow is also moved every day by piste grooming. These mechanisms can lead to the absorption of organic compounds on mineral particles, potentially altering ice nucleation activity in both directions (Boose et al., 2019) through absorption and redox reactions on their surface. In the aged snow samples from the piste, EDX showed one rather different particle from the others, with a main composition of O, Bi, Cl, and Na. The chlorine and sodium had a similar ratio and it likely originated from de-icing salt that was transported onto the piste by machinery and absorbed on the particle. The origin of the bismuth is unclear. It is either of natural origin, or was brought into the system by anthropogenic influences since it is used in certain weather resistant pigments.

Particles of biological origin were found in the natural and aged snow samples. Some of these were clearly from plant material, while others were unidentified. A plant fibers’ ice nucleation ability can be attributed to cellulose and ice nucleating macromolecules. Cellulose covers a wide range of ice nucleation activity (−16 to −29°C) similar to mineral dust samples (Hiranuma et al., 2015, 2019). Ice nucleating macromolecules originating from plants can be washed off and have been found to nucleate ice at around −17°C for birch (Pummer et al., 2012, 2015; Augustin et al., 2013; Felgitsch et al., 2018) and to be composed of polysaccharides (Dreischmeier et al., 2017). Such macromolecules suspended in water are not easily detected, but may be the origin of the film that covered the river, pump, and artificial snow samples as well as the agglomerations of carbon rich microparticles seen on the other samples, in combination with other soluble organic compounds and microbes. All of these can act as efficient INPs. The DOC values suggest a dilution and selection effect of these in the river, pump and artificial snow samples. Aged and fresh snow samples showed the highest amount of DOC, indicating that they are most rich in these compounds. They may account for some of the higher than –15°C freezing events of these samples observed in the DRINCZ experiment along with microbial activity.

The aqueous chemistry supported most of the observations from SEM/EDX measurements and was within the expected range for alpine water systems (Füreder et al., 2001). Ammonium concentrations were highest in aged snow. Since part of the piste in Obergurgl is used as pasture fertilized with cow manure which is high in ammonium by the local farmers in the summertime, diffusion processes might have transported some of the ammonium to the upper snow layers. Another potential source could be microbial and enzymatic activity, as NH_4_^+^ is produced by some microbes through nitrogenase (Burgess and Lowe, 1996; Hoffman et al., 2014), pointing to microbial activity in aged snowpack. Ammonium was also found to activate ice nucleation ability in montmorillonite minerals (Salam et al., 2007).

Ice nucleating particles of non-microbial nature were found in abundance in all samples and their change in composition was tracked through the water cycle of artificial snowmaking. The largest share was composed of mineral particles that undergo selection and dilution processes, leading to a shift in their composition. Chemical weathering processes can also alter their ice nucleation ability (Sullivan et al., 2010; Zolles et al., 2015; Boose et al., 2019). The same processes apply to the particles of biogenic origin. The DRINCZ results showed a decrease in the ice nucleation ability after heat treatment for all samples. This is an indicator that biological material influenced all different kinds of INPs, likely by absorption. Whether its nature is that of living microbes, their fragments, or from proteinaceous plant material is difficult to discern. The transport mechanisms of active microbes are the same as those for other particles, but since they can multiply under the right conditions, it can lead to an enrichment of ice nucleation active communities in some places. Temperature and nutrient availability play an important role for this effect, thus there might be a change in activity over the course of a year.

### Biology Drives Highly Active Ice Nucleation in Snow and Water Samples

The highest concentration of INPs at high temperatures, but also the largest heterogeneities and highest change in T50 (ΔT50) occurred in the aged piste snow, with two of the three samples showing higher activity than any other sample. While aged piste snow had the highest unheated IN-activity and had a high relative share of ice-nucleation containing bacteria, it also had the lowest absolute number of ice-nucleation protein containing bacteria per mL (only half of the lowest fresh snow sample). Although 16S qPCR results cannot be taken as absolute numbers since multiple copies can be present in one bacterial cell (Větrovský and Baldrian, 2013), it has also been shown that cell count does not correlate with ice-nucleation effectivity (Joly et al., 2013). These results suggest that bacterial strains with high ice-nucleating activity at high temperatures were introduced into the piste snow. Based on the blast results against the ice-nucleating database, these bacteria likely belong to *Pseudomonas* and *Pantoea* (Figure 8).

The onset ice-nucleation temperatures of biological nucleators like *P. syringae* range from −3 to −10°C for different strains (Joly et al., 2013). Generally, the protein structures found on ice nucleation active bacteria fall into three categories: the first is able to nucleate above −4.4°C and is sensitive to organic solvents, the second nucleates ice between −4.8 and −5.7°C, and the third, which is the most commonly found, nucleates at −7.6°C or cooler (Turner et al., 1990; Wex et al., 2015; Häusler et al., 2018). Mortazavi et al. (2008) isolated a range of bacterial strains from snow that were IN-active at temperatures between −12.9°C (i.e., *Xanthomonas*) and −17.5°C (*Microbacterium imperiale*, *Microbacterium arborescens*), which is within the range of IN-active minerals like kaoline and montmorillonite. Hill et al. (2014) found biological INPs that contributed 85% of INPs active at −10°C in snow samples. The environment, especially soil minerals, harbors a reservoir of biological nano-ice-nucleation molecules (O’Sullivan et al., 2015; Augustin-Bauditz et al., 2016), which could have been introduced into aged piste snow by grooming. The longer exposure to air, and therefore, potential settlement of ice-nucleation active organisms, could be another source of the active ice-nucleators (e.g., from surrounding soil and plant surfaces), as aged piste snow revealed the highest number of hits to an alpine air database (see Supplementary Figures S4, S5; Els et al., 2019). Although piste snow was not sampled before the aging occurred, the very efficient and high concentration of INP in the aged snow suggests the introduction of INP from different sources due to anthropogenic activity.

The heated piste snow was the most active sample at low temperatures, indicating the role of introduced chemical, plant residual or inorganic components such as soil minerals, chemical components of ski wax, ski alloy or exhaust aerosols from grooming machines. These components might also act as ice nuclei at high temperatures or enhance ice-nucleation protein onset temperature (Watanabe and Watanabe, 1994).

The deactivation of the most efficient INPs after heating indicates that they were of biological nature or thermally labile. Previous studies of both precipitation and water samples have shown a strong impact of heating on INP efficiency and concentration (Schnell and Vali, 1975; Christner et al., 2008a; Hill et al., 2014; Wilson et al., 2015; Irish et al., 2017). Consistent with the observations of Christner et al. (2008a), all of the samples lost their ability to nucleate ice at T ≥ −9°C except for the aged piste snow sample. This may be related to a sample preparation bias during the experiment. These samples were placed at the center of the well plate (see Supplementary Figure S9) and likely never reached the 95°C needed to destroy the INP present in the aliquots.

In our study, the source for artificial snow was the river water, which had the third-highest unheated ice-nucleation activity and several hits to *Pseudomonas* strains (see Supplementary Figure S8). Despite featuring a different composition of ice-nucleation protein containing bacteria that were not as active as piste samples, artificial snow and its sources contained ice-nucleation active bacteria, and had higher absolute ice-nucleation protein containing 16S numbers. Artificial snow and its sources displayed very similar unheated IN temperatures and concentration curves and ΔT50 values, which suggests that they had the same water source and same timeframe of collection. This indicates that quantifying the INP concentration of the source water in artificial snow production might be a useful tool to elucidate and optimize the temperature thresholds for snow making.

The water used for artificial snow production usually undergoes a UV-treatment in the Obergurgl ski resort, as this is mandatory in the federal state of Tyrol. Here, we chose not to apply this treatment in order to better trace microorganisms from their source environments to the artificial snow and to make the study more representative for ski resorts in Austria and globally where UV is not used. We expect the ice-nucleation activity to be comparably high after UV-treatment, as bacterial ice-nucleation proteins are located on the outer membrane (Kozloff et al., 1991), offering a suitable nucleation surface structure even when bacterial metabolism is disabled (Maki et al., 1974). UV-purification disrupts the DNA-strands, inhibiting further proliferation of the cells, however, IN-activity was shown to be unaffected by atmospheric UV-radiation levels (de Araujo et al., 2019).

The natural snow samples showed the lowest activities of all samples and extended below the previously reported INP concentrations for precipitation (Petters and Wright, 2015). The lower than previously reported values may be due to the sampling site being located in the heart of the Alps where upstream mountains may have removed the more efficient INPs from the air mass, as reported by Stopelli et al. (2015). The relative abundance of ice-nucleation protein containing bacteria was substantially lower in fresh snow than in the piste and artificial snow. In absolute numbers, the onset temperatures were highest in fresh snow, but concentrations were low. The fresh snow samples showed the least sensitivity to heating, indicating that the majority of the INPs in the samples were of non-microbial nature. Thus, if taken as a baseline for naturally occurring ice nucleation potential, the physically expressed ice-nucleation activity, but also the relative and absolute share of biological ice nucleators is higher in all other investigated sources (pond, river, artificial, piste) than in freshly fallen snow.

### Ecological Implications of the Investigated Samples

Our results illustrate the potential of artificial snow production to alter biological and chemical to adjacent high alpine soil and water ecosystems. When melting occurs at warmer temperatures, snowpack percolation of meltwater and subsequent supply to the underlying soil ecosystem is likely (Lazzaro et al., 2015b). Our results imply that a higher supply of SO_4_^2–^, Ca^2+^, Mg^2+^, and K^+^, and a lower supply of Cl^–^, NO_3_^–^, DN, and DOC can be introduced to ecosystems from artificial snow. The excess addition of cations by artificial snow might affect the pH and lower the cation exchange capacity, an important parameter for nutrient supply, on soil mineral particles with high inputs (Barak et al., 1997; Liang et al., 2006).

64 bacterial (4.7% of total genera) and 5 fungal (1.5% of total) genera were unique to the artificial snow. These might originate from biofilms within the pipe system of the snow cannon or the air intake (approximately 17 m^3^ s^–1^) at the snow production process, thus there is a potential for introducing different species. Non-native genetic material can be distributed to a natural high alpine ecosystem, and with the fast reproduction cycles of microbes, extracellular environmental DNA could be incorporated and multiplied during cell reproduction. In fact, uptake of extracellular DNA and natural transformation is an established way for microorganisms to acquire new genetic information (Lorenz and Wackernagel, 1994; Chen and Dubnau, 2004; Nielsen et al., 2007).

The lowest bacteria to fungi rate (and at the same time the highest number of fungal genera) was found in piste snow. This could be due to the introduction of fungal spores by humans or settling from the air. The artificial snow contained 8.1% (i.e., 27) fungal genera in common with fresh and piste snow, compared to only 1.5% unique fungal genera common in artificial snow, pond and river water. This indicates an introduction of fungi in the artificial snow production process, likely from air, as approx. 60,000 m^3^ of air per hour are needed for artificial snow production. Artificial snow had a multiple times higher hit rate on an alpine air fungal database, indicating an uptake by pressurized air as a likely source (see Supplementary Figures S4, S5).

## Conclusion

In this study, we tracked the flow of INPs in the water cycle of an alpine ski resort. We analyzed microbial abundances and composition, dissolved and particulate chemical composition, and ice nucleation activity of various snow and water samples that represent steps in the water cycle of artificial snow production. To exclude confounding factors, such as catchment geology or meteorological features, this was done in triplicate at a single field site.

The results provided insight on the flow of biological and inorganic particles as well as dissolved ions in this mixed natural and artificial environment and improved our understanding of the introduction of new INPs in this system and how they change in different sample types.

Inorganic particles where found in all samples. The samples that originated from the river water all had a distinctive composition high in Ca, C, and S that could be attributed to the river as a source. Sedimentation of particles in the storage pond did not affect the ice nucleation since there was no significant difference between river water and the pumping station. The particle load of a glacial fed river varies strongly over the course of year and future research carried over an entire year could show how ice nucleation activity is impacted by this. The fresh natural snow samples and the piste snow samples – which consisted mostly of aged natural snow – had a different mineral composition than the river samples as more particles showed to be rich in Si and O, likely from quartz and feldspar, as is common for dust in aerosol samples.

For all samples, the ice nucleation activity changed to lower T50 after a heat treatment of 95°C, indicating that the most active INPs seem to be of microbial origin, or from other heat labile compounds. Microbial INPs seem to be most likely since 97% similarity hits for organisms with ice nucleation active proteins were found in all samples.

This indicates that biological components are driving the ice-nucleation activity in artificial snow and its sources and, fresh and piste snow, with the most active components found in natural piste snow. These were likely introduced by grooming or the prolonged exposure to surrounding sources. Freshly fallen natural snow had the lowest biological inorganic IN-activity of all investigated samples.

Closest database hits to different strains of *P. syringae* (and other known ice nucleators *Erwinia*, *Pantoea*, *Xanthomoas*) were present in artificial snow and piste snow.

Close to surface air-microbes, especially fungi, that were not present in the pond or river water or natural snow samples, were introduced to artificial snow through the uptake of high volumes of pressurized air. This constitutes a potentially new pathway of microbe introduction to artificial snow, suggesting that the rate of incorporation of dry aerosols into artificial snow is greater than what is scrubbed by natural snowfall.

Artificial snow production might serve as an easily controllable model to collect these microbes.

Further research concerning the role of artificial snow chemistry and microbiology on the adjacent soil and water ecosystems in high alpine settings would be highly relevant to understand the ecological impact of large-scale distribution of artificial snow.

## Data Availability Statement

The datasets generated for this study can be found in the NCBI – Accession: PRJNA 534428 ID: 534428.

## Author Contributions

PB and NE contributed to the data analysis and manuscript in equal parts, and are therefore both considered first authors. PB and HG designed the study. PB collected the samples, did SEM and EDX analyses, and wrote the manuscript. HG and BS provided funding and contributed to the manuscript. NE conducted the molecular biological lab, bioinformatics, aquatic chemistry, and statistical analyses, and wrote the manuscript. RD conducted the DRINCZ measurement and contributed to the manuscript. CL counseled the molecular biology analysis and contributed to the manuscript. KW helped with the SEM and EDX analyses.

## Conflict of Interest

The authors declare that the research was conducted in the absence of any commercial or financial relationships that could be construed as a potential conflict of interest.

## Footnotes

1 www.ncbi.nlm.nih.gov
