# Supplementary material for: Assessment of Artificial and Natural Transport Mechanisms of Ice Nucleating Particles in an Alpine Ski Resort in Obergurgl, Austria
Source: Front Microbiol. 2019 Oct 4;10:2278. doi: 10.3389/fmicb.2019.02278 (PMC6788259; doi:10.3389/fmicb.2019.02278)
Supplement: Supplementary file 1 [file Data_Sheet_1.pdf]

1 Supplementary Information

SI-Table 1: ANOSIM, ADONIS and pairwise PERMANOVA for Sample Types

SampleType	Bacteria					Fungi				
**ANOSIM (bray)**	R=0.962	**p=0.001**				R=0.7498	**p=0.001**			
**ADONIS (bray)**	Df=5	MeanSqs: 0.060	F-Model: 13.153	R^2=0.868	**p=0.001**	Df=5	MeanSqs: 0.5682	F-Model: 2.6965	R^2=0.5742	**p=0.001**
**Pairwise (bray) PERMANOVA**	**Df**	**F-Model**	**R^2**	**p**	**p adj. (holm)**	**Df**	**F-Model**	**R^2**	**p**	**p adj. (holm)**
**piste vs fresh**	8	3.8334	0.3538	**0.010**	**0.050**	8	1.9879	0.2212	**0.013**	0.052
**piste vs pond**	6	13.4051	0.7283	**0.034**	0.102	6	4.7962	0.4895	**0.031**	0.093
**piste vs artificial**	5	18.3371	0.8209	0.100	0.200	5	2.2994	0.3650	0.100	0.182
**fresh vs pond**	9	18.9790	0.7035	**0.005**	**0.030**	9	5.4742	0.4063	**0.004**	**0.024**
**fresh vs artificial**	8	18.8756	0.7295	**0.010**	**0.050**	8	2.4731	0.2612	**0.010**	**0.050**
**pond vs artificial**	6	2.1282	0.2986	0.141	0.200	6	1.7365	0.2578	0.091	0.182

All values calculated from the thirdroot transformed, 97% most common OTUfiltered, genus level datasets
Pairwise PERMANOVA(bray): samples fresh snow 1 & 2 resp. pond and river were grouped together to “fresh” and “pond” to lower number of comparisons, holm p-value correction was applied

SI-Table 2: Relative abundance of ten most abundant bacterial phyla and fungal classes

Bacteria	Aged Piste Snow	Artificial snow	Fresh snow1	Fresh snow2	Pond pump	River
**Acidobacteria**	8.87%	3.34%	8.51%	10.06%	0.32%	3.58%
**Actinobacteria**	10.05%	6.79%	13.92%	14.69%	1.19%	6.98%
**Bacteriodetes**	4.32%	6.16%	6.45%	4.99%	1.88%	6.49%
**Chloroflexi**	1.27%	1.14%	6.85%	9.16%	0.10%	0.85%
**Cyanobacteria**	13.91%	0.65%	26.87%	31.34%	0.31%	0.82%
**Firmicutes**	2.48%	1.29%	1.49%	1.32%	0.32%	0.48%
**Patescibacteria**	0.03%	10.39%	0.04%	0.03%	5.82%	36.36%
**Planctomycetes**	0.38%	4.93%	0.44%	0.51%	2.24%	1.50%
**Proteobacteria**	58.01%	64.79%	34.44%	27.03%	87.31%	39.72%
**Verrucomicrobia**	0.68%	0.52%	0.98%	0.87%	0.55%	3.21%

Fungi	Aged Piste Snow	Artificial snow	Fresh snow1	Fresh snow2	Pond pump	River
**Aaricomycetes**	0.00%	0.86%	1.19%	1.21%	0.78%	1.16%
**Chytridiomycetes**	0.00%	0.00%	0.00%	0.00%	0.00%	54.65%
**Dothideomycetes**	14.67%	13.66%	8.65%	12.53%	0.00%	6.98%
**Eurotiomycetes**	13.24%	11.69%	20.07%	18.52%	0.00%	0.00%
**Lecarnomycetes**	34.78%	32.41%	48.07%	30.27%	0.00%	0.00%
**Leotiomycetes**	4.91%	5.91%	7.47%	10.78%	0.41%	5.81%
**Rhizophydiomycetes**	0.00%	1.78%	0.00%	0.00%	14.08%	4.65%
**Sordariomycetes**	1.28%	2.08%	3.14%	5.87%	0.00%	0.00%
**Tremellomycetes**	4.99%	0.81%	1.97%	8.00%	0.00%	4.65%
**unidentified**	26.12%	3079%	9.44%	12.81%	84.74%	22.09%

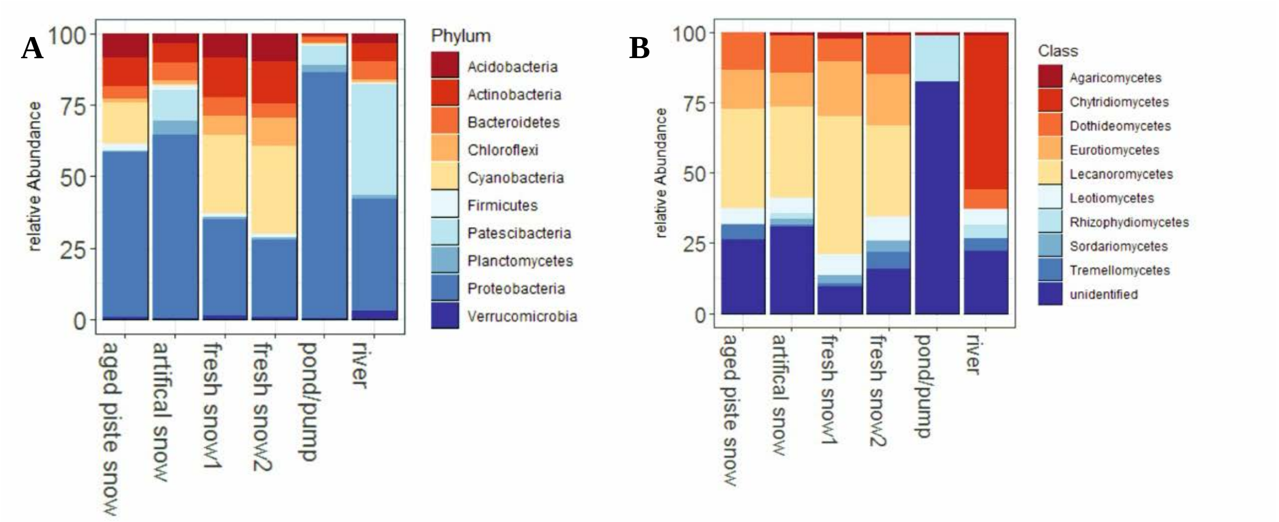

SI-Figure 1: A) % relative Abundance of ten most abundant bacterial phyla and B) fungal classes for the different sample types

The 10 most abundant phyla displayed high differences between the sample types. *Proteobacteria* were dominant for all samples, with the most prominent relative abundance in the pond/pump water (87.31%) and the lowest in fresh snow 2 (27.03%). Most prominently *Cyanobacteria* ranged from 13.9% in piste snow to 31.34% in fresh snow and only 0.31% to 0.82% in pond, river and artificial snow, predominantly driving differences to artificial snow and its sources. *Patescibacteria* were especially driving the difference of river bacterial communities (36.36%) compared to all other sample types (reaching 10.39% in artificial snow and 0.04% in natural snow). Further *Actinobacteria* accounted to 14.69% (fresh snow 2) to 10.05% (piste) in natural snow samples compared to 1.19% to 6.98% in pond/pump resp. river water. *Firmicutes* were highest in piste snow (2.48%). *Planctomycetes* hat the highest abundance in artificial snow (4.93%).

The 10 most abundant fungal classes show a similar composition in fresh snow 1&2, piste snow and also artificial snow with a high share of *Lecarnomycetes* (48.07%-30.27%) and *Eurotiomycetes* (11.69%-20.07%) and *Dothideomycetes* (8.65%-14.67%), that are absent in pond/pump and river water (i.e *Lecarnomyetes*, *Eurotiomycetes*). Pond/pump shows a high share of *unidentified* (84.74%) and *Rhizophydiomcetes* (14.08%), while river water is high in *Chytridiomycetes* (54.65%) that are absent in all other samples.

SI-Table 3: Kruskal-Wallis Test statistics for Sample Types group comparison, asteriks (*) mark significant values

Kruskal Wallis Test ~Sample Type	p-value (alpha=0.05)	X^2	df
**Chao1 (bacteria)**	P=0.03*	X^2=12.3235	5
**Shannon (bacteria)**	P=0.02*	X^2=13.2353	5
**InvSimpson (bacteria)**	P=0.04*	X^2=11.8529	5
**Chao1 (fungi)**	P=0.03*	X^2=12.2059	5
**Shannon (fungi)**	P=0.17	X^2=7.6765	5
**InvSimpson (fungi)**	P=0.16	X^2=7.8529	5
**16S mL-1**	P=0.03*	X^2=12.4412	5
**18S mL-1**	P=0.13	X^2=8.5588	5
**16S to 18S ratio**	P=0.03*	X^2=12.1471	5
**DOC**	P=0.02*	X^2=13.4444	5
**DN**	P=0.01*	X^2=15.5882	5
**Cl**	P=0.18	X^2=7.5882	5
**NO3**	P=0.01*	X^2=15.3268	5
**SO4**	P=0.02*	X^2=13.0319	5
**Na**	P=0.56	X^2=3.9477	5
**NH4**	P=0.02*	X^2=13.5656	5
**K**	P=0.02*	X^2=12.8693	5
**Mg**	P=0.03*	X^2=12.4663	5
**Ca**	P=0.01*	X^2=14.3822	5

SI-Table 4: Dunns Post Hoc Pairwise Test, no p-value correction applied (when applied no significant p values were obtained), asteriks mark significant p values

Comparisons	Chao1* (bact)	Shannon * (bact)	InvSimp (bact)*	Chao1* (fungi)	Shannon (fungi)	InvSimp (fungi)
**aged piste snow - artifical snow**	0.003*	0.019*	0.051	0.012*	0.151	0.099
**aged piste snow - fresh snow1**	0.220	0.151	0.431	0.072	0.431	0.431
**artifical snow - fresh snow1**	0.024*	0.151	0.072	0.220	0.195	0.132
**aged piste snow - fresh snow2**	0.029*	0.061	0.334	0.099	0.365	0.274
**artifical snow - fresh snow2**	0.195	0.303	0.114	0.172	0.085	0.029*
**fresh snow1 - fresh snow2**	0.132	0.303	0.398	0.431	0.303	0.220
**aged piste snow - pond/pump**	0.274	0.220	0.085	0.000*	0.029*	0.085
**artifical snow - pond/pump**	0.016*	0.002*	0.001*	0.132	0.195	0.465
**fresh snow1 - pond/pump**	0.431	0.035*	0.061	0.029*	0.043*	0.114
**fresh snow2 - pond/pump**	0.099	0.010*	0.035*	0.019*	0.012*	0.024*
**aged piste snow - river**	0.010*	0.022*	0.057	0.101	0.072	0.057
**artifical snow - river**	0.358	0.292	0.335	0.380	0.233	0.252
**fresh snow1 - river**	0.039*	0.101	0.072	0.404	0.091	0.072
**fresh snow2 - river**	0.165	0.181	0.101	0.358	0.044*	0.022*
**pond/pump - river**	0.030*	0.005*	0.005*	0.137	0.451	0.272

Comparisons	16S*	18S	16S:18S*	DOC*	DN*	Cl	NO3*
**aged piste snow - artificial snow**	0.085	0.072	0.008*	0.045*	0.233	0.147	0.053
**aged piste snow - fresh snow1**	0.274	0.043*	0.152	0.314	0.073	0.343	0.001*
**artificial snow - fresh snow1**	0.024*	0.398	0.085	0.113	0.015*	0.259	0.073
**aged piste snow - fresh snow2**	0.334	0.303	0.334	0.404	0.233	0.286	0.010*
**artificial snow - fresh snow2**	0.036*	0.173	0.024*	0.073	0.073	0.314	0.233
**fresh snow1 - fresh snow2**	0.432	0.115	0.274	0.404	0.233	0.436	0.233
**aged piste snow - pond**	0.013*	0.220	0.002*	0.003*	0.073	0.008*	0.187
**artificial snow - pond**	0.196	0.013*	0.334	0.147	0.233	0.085	0.233
**fresh snow1 - pond**	0.002*	0.006*	0.036*	0.012*	0.002*	0.022*	0.015*
**fresh snow2 - pond**	0.004*	0.099	0.008*	0.006*	0.015*	0.031*	0.073
**aged piste snow - river**	0.198	0.452	0.198	0.011*	0.033*	0.079	0.428
**artificial snow - river**	0.452	0.182	0.198	0.224	0.116	0.319	0.052
**fresh snow1 - river**	0.101	0.138	0.452	0.033*	0.001*	0.147	0.002*
**fresh snow2 - river**	0.125	0.404	0.293	0.020*	0.006*	0.183	0.011*
**pond - river**	0.233	0.252	0.125	0.428	0.294	0.224	0.164

Comparisons	SO4*	Na	NH4*	K*	Mg*	Cl*
**aged piste snow - artificial snow**	0.012*	0.038*	0.053	0.073	0.062	0.197
**aged piste snow - fresh snow1**	0.259	0.286	0.085	0.286	0.373	0.197
**artificial snow - fresh snow1**	0.053	0.113	0.404	0.022*	0.031*	0.044*
**aged piste snow - fresh snow2**	0.209	0.113	0.085	0.187	0.343	0.165
**artificial snow - fresh snow2**	0.073	0.286	0.404	0.010*	0.026*	0.034*
**fresh snow1 - fresh snow2**	0.436	0.259	0.500	0.373	0.468	0.452
**aged piste snow - pond**	0.004*	0.166	0.000*	0.045*	0.026*	0.034*
**artificial snow - pond**	0.343	0.209	0.038*	0.404	0.343	0.165
**fresh snow1 - pond**	0.022*	0.343	0.022*	0.012*	0.012*	0.004*
**fresh snow2 - pond**	0.031*	0.404	0.022*	0.005*	0.009*	0.003*
**aged piste snow - river**	0.006*	0.097	0.006*	0.079	0.033*	0.051
**artificial snow - river**	0.319	0.386	0.147	0.457	0.319	0.192
**fresh snow1 - river**	0.028*	0.213	0.103	0.028*	0.016*	0.008*
**fresh snow2 - river**	0.038*	0.414	0.103	0.014*	0.014*	0.006*
**pond – river**	0.457	0.332	0.294	0.457	0.457	0.500

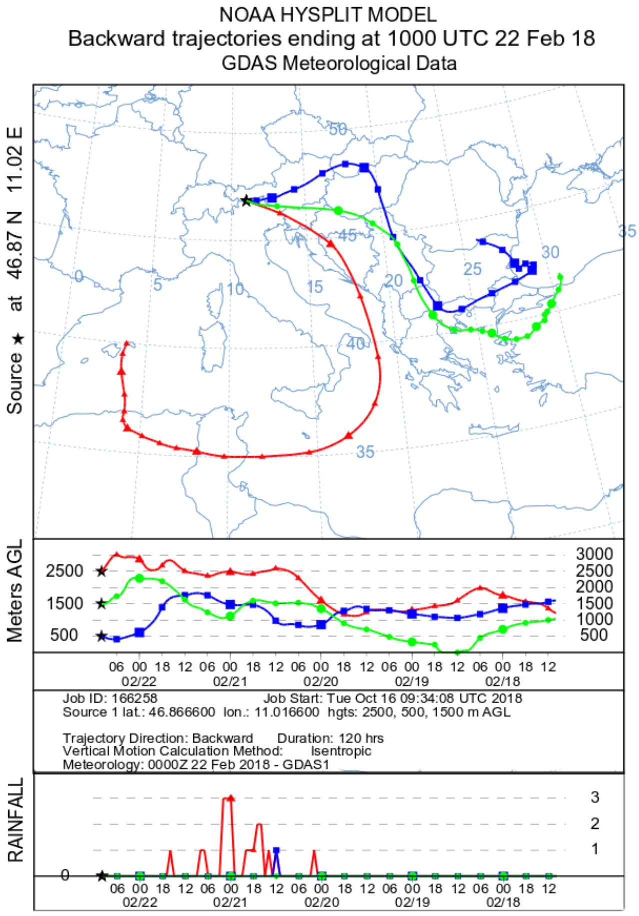

SI-Figure 2: 120hrs Hysplit backtrajectory of air masses for the time of snow precipitation in Obergurgl that was sampled as fresh snow and aged piste snow for 2500m, 1500m and 500m AGL arrival height

SI-Table 5: Sequence Statistics

	Bacteria	***Fungi***
#raw sequences per sample	25268+/-9307	31183+/- 12789
OTUs raw dataset	5947	1476
OTUs after blank removal	5874	992
Most abundant 97% OTUs	3095	940
Merged on genus level	958	326
Rarefied	956	--

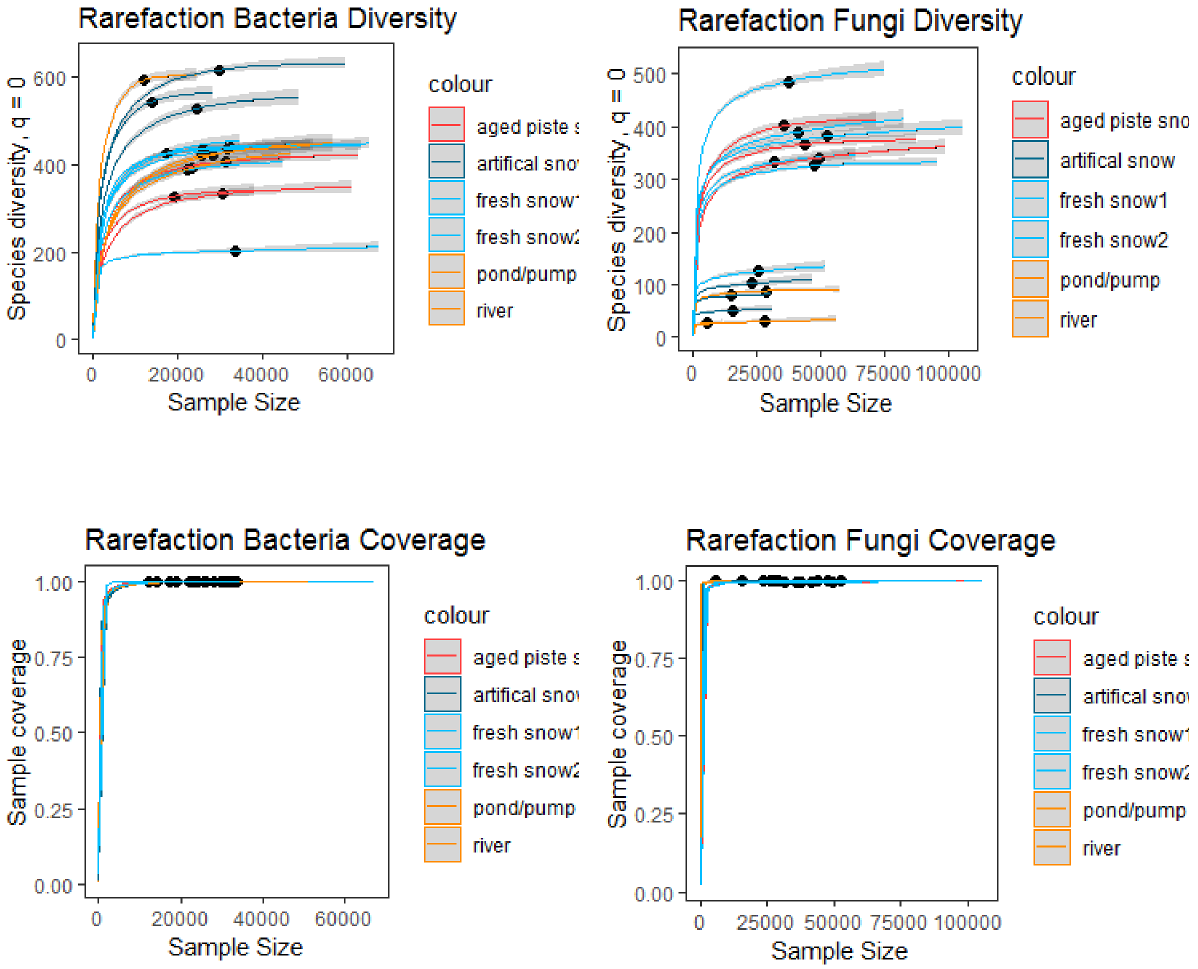

SI-Figure 3: Diversity and Coverage Rarefaction for Bacteria and Fungi sequences

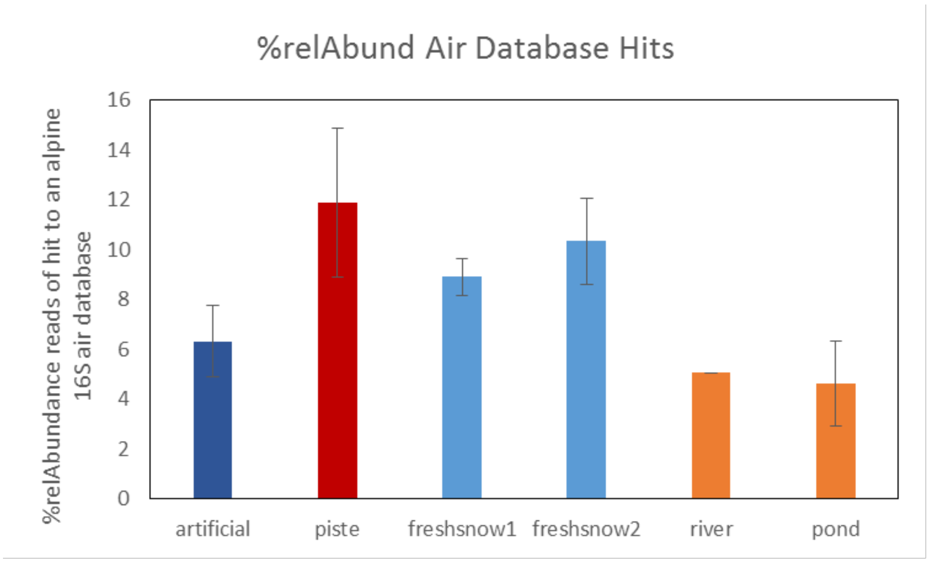

SI-Figure 4: Relative abundance of total reads for each sample type being a hit to an alpine air 16S database, air database from alpine air sequences of Els et al., 2019 and Els et al., (in review)

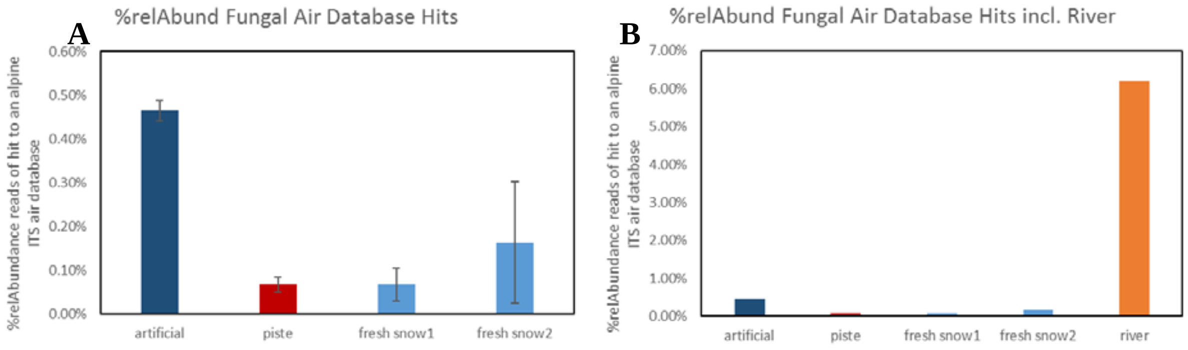

SI-Figure 5 Relative abundance of total reads for each sample type being a hit to an alpine air ITS database, A) only snow samples, B) including the river sample, air database from alpine air sequences of Els et al., 2019 and Els et al., (in review)

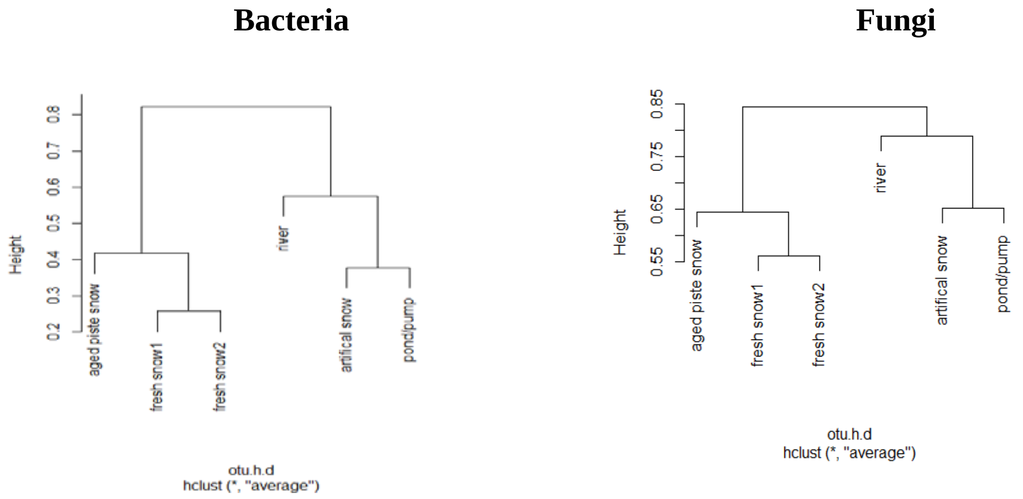

SI-Figure 6: Cluster Dendrogram, hellingertransf. bray, average clustering of merged sample types

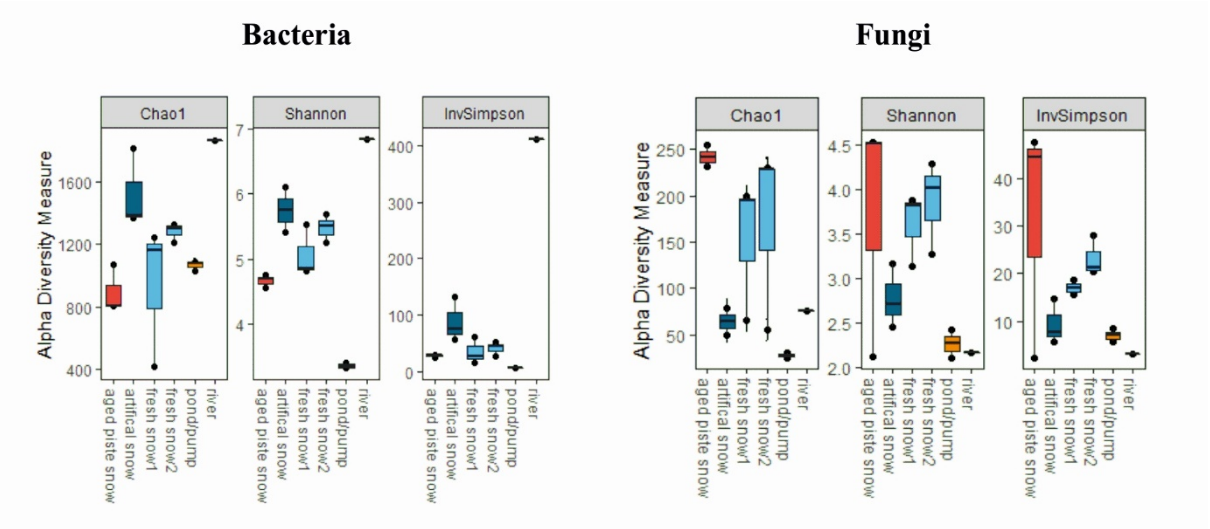

SI-Figure 7: Alpha diversity metrics

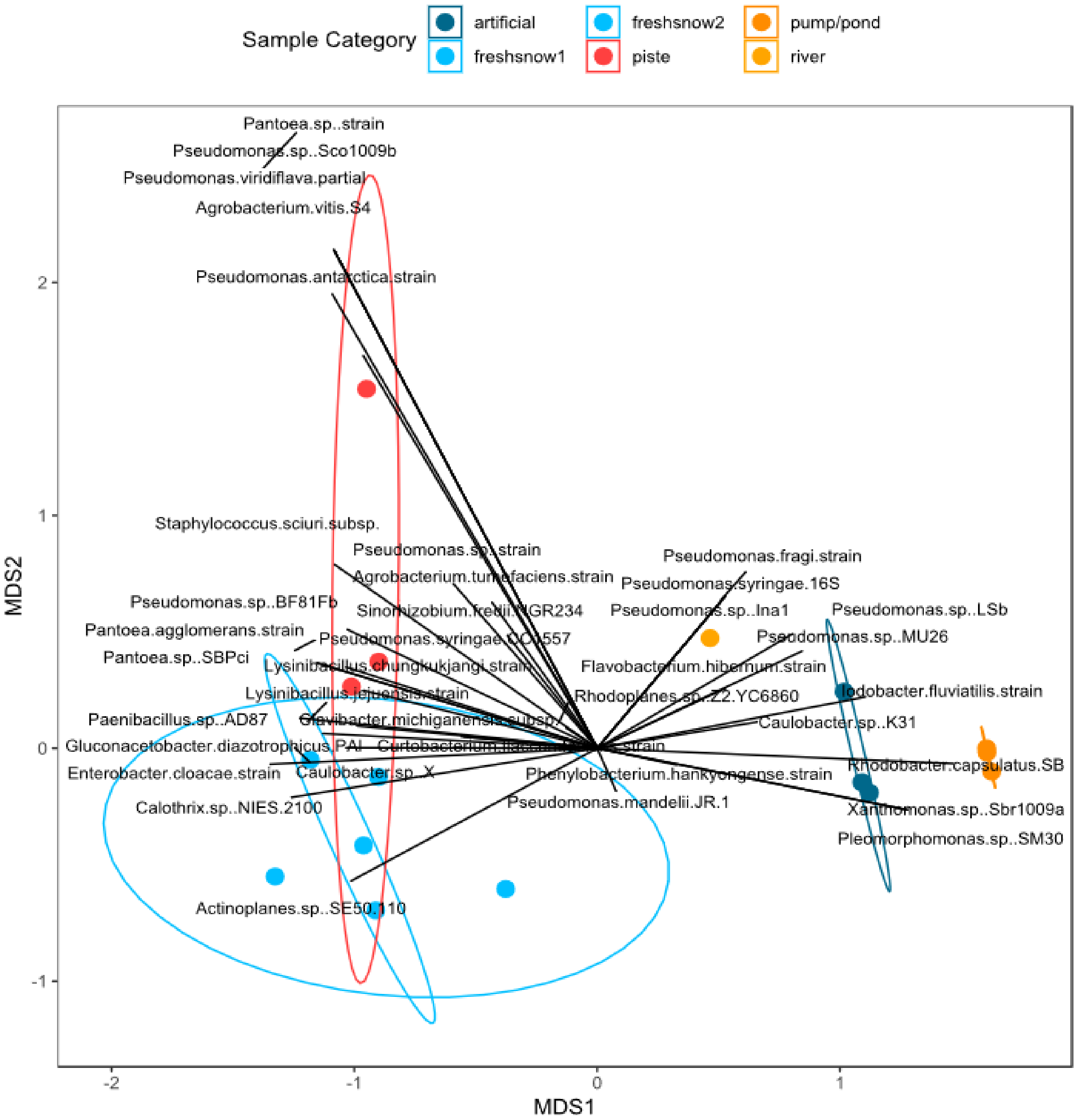

SI-Figure 8: NMDS of closest hits to ice nucleation database on strain level

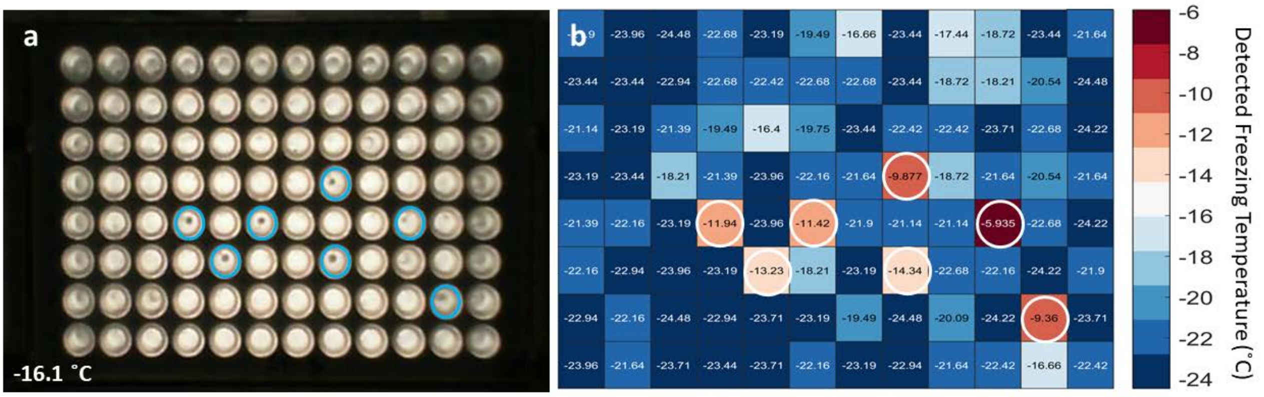

SI-Figure 9: DRINCZ setup with the highest freezing temperature clustering in the middle of the sample tray indicating uneven heat distribution to the aged piste snow sample.

Sample	DOC µg/L	DN µg/L	Cl^- mg/L	NO_3^+ mg/L	$SO4^{2+}$ mg/L	Na^+ mg/L	$NH4^+$ mg/L	K^+ mg/L	Mg^{2+} mg/L	Ca^{2+} mg/L
River #1_1	466	214	0.332	0.198	65.000	1.366	0.009	4.514	7.658	35.000
River #1_2	303	152	0.230	0.139	48.000	1.016	0.006	3.412	5.865	29.000
Natural Snow #1_1	611	547	0.488	0.499	0.554	0.311	0.036	0.043	0.030	0.456
Natural Snow #1_2	598	563	0.256	0.523	0.519	0.134	0.041	0.037	0.026	0.359
Natural Snow #1_3	2672	1074	6.687	0.572	1.255	3.943	0.065	0.097	0.281	0.552
Natural Snow #2_1	729	514	0.132	0.464	0.524	0.042	0.031	0.035	0.024	0.381
Natural Snow #2_2	693	424	2.929	0.370	1.017	1.867	0.054	0.045	0.057	0.524
Natural Snow #2_3	763	502	2.143	0.434	0.767	1.269	0.044	0.059	0.115	0.398
Artificial Snow #1_1	553	330	0.411	0.281	45.000	1.321	0.042	3.668	5.132	17.986
Artificial Snow #1_2	576	309	0.444	0.272	45.000	1.231	0.049	3.652	5.180	17.417
Artificial Snow #1_3	470	301	0.286	0.277	54.000	1.262	0.015	4.303	6.132	18.798
Aged Piste Snow #1_1	834	365	0.612	0.202	0.577	0.367	0.088	0.076	0.100	0.654
Aged Piste Snow #1_2	656	371	0.619	0.196	0.457	0.355	0.091	0.064	0.086	0.524
Aged Piste Snow #1_3	775	367	0.551	0.193	0.494	0.334	0.092	0.066	0.100	0.556
Pumping Station #1_1	398	270	0.222	0.258	53.000	1.045	0.005	3.702	6.003	33.000
Pumping Station #1_2	299	263	0.226	0.254	52.000	1.043	0.000	3.691	5.989	33.000
Pumping Station #1_3	324	262	0.203	0.248	52.000	1.034	0.000	3.693	5.983	33.000

SI-Table 6: Water chemistry values of snow and water samples
